# Evaluation of Anticancer and Cytotoxic Effects of Genistein on PC3 Prostate Cell Line under Three-Dimensional Culture Medium

**DOI:** 10.52547/ibj.3711

**Published:** 2022-11-20

**Authors:** Seyedeh Masoumeh Khamesi, Mehdi Salehi Barough, Jamil Zargan, Mohsen Shayesteh, Nooshin Banaee, Ashkan Haji Noormohammadi, Hani Keshavarz Alikhani, Mohsen Mousavi

**Affiliations:** 1 Department of Medical Radiation Engineering, Central Tehran Branch, Islamic Azad University, Tehran, Iran;; 2 Department of Biology, Faculty of Basic Science, Imam Hossein University, Tehran, Iran;; 3 Department of Physics, Imam Hossein University, Tehran, Iran

**Keywords:** Apoptosis, Genistein, Three-dimensional cell culture

## Abstract

**Background:**

Prostate cancer is a major cause of disease and mortality among men. GNT is an isoflavone found naturally in legumes. Isoflavones, a subset of phytoestrogens, are structurally similar to mammalian estrogens. This study aimed to evaluate the anticancer and cytotoxic effects of GNT on PC3 cell line under 3D culture medium.

**Methods:**

The 3D culture was created by encapsulating the PC3 cells in alginate hydrogel. MTT assay, neutral red uptake, comet assay, and cytochrome C assay were used to study the anticancer and cytotoxic effects of GNT at 120, 240, and 480 μM concentrations. Also, NO, catalase, and GSH levels were determined to evaluate the effect of GNT on the cellular stress. The culture medium was used as the negative control.

**Results:**

GNT reduced the production of cellular NO and increased the production of catalase and glutathione, confirming the results of the NO test. Evaluation of the toxicity effect of GNT at the concentrations of 120, 240, and 480 μM using comet assay showed that this chemical agent induces apoptosis in PC3 cells in a dose-dependent manner. As the level of cytochrome C in PC3 cells treated with different concentrations of GNT was not significantly different from that of the control, GNT could induce apoptosis in PC3 cells through the non-mitochondrial pathway.

**Conclusion:**

The findings of this study disclose that the anticancer effect of GNT on PC3 cells under 3D culture conditions could increase the effectiveness of treatment. Also, the cell survival rate is dependent on GNT concentration.

## INTRODUCTION

Cancer is the second leading cause of mortality after heart disease in the world and has affected many people for many years^[^^1^^-^^3^^]^. Globally, PCa is the most common malignancy and a major cause of death in men, and its incidence, particularly in developing countries, is rising every year^[^^4^^-^^6^^]^.

Surgery, chemotherapy, and radiation are all conventional cancer therapies that play a limited but crucial role in the overall treatment of PCa. Cancer therapy through combining radiation and intermediates with distinct molecular properties have been developed to boost the efficiency of treatment, thus improving survival outcomes. Recently, 3,3’-diindolylmethane, indole-3-carbinol, curcumin, epigallocatechin-3-gallate, resveratrol, and GNT have been investigated for their anticancer effects. Combined treatment with these compounds has been demonstrated to decrease patient's radiation dosage and reduce the systemic toxicity of chemotherapy or radiation^[^^7^^]^. 

GNT (C_15_H_10_O_5_) is a naturally occurring chemical constituent that structurally belongs to a class of compounds known as isoflavones. It is abundantly found in legumes, particularly soy; one gram of soybean seed contains 500 μg of GNT^[^^8^^]^. A large body of evidence has indicated the antitumor and antiangiogenic properties of GNT on various types of human cancers, including PCa^[^^9^^-^^11^^]^. One of the most well-known functions of soy isoflavones in PCa is the prevention of epithelial cell growth^[^^12^^]^. Findings have demonstrated that GNT inhibits the cell growth by inducing the G2/M cell cycle arrest^[^^13^^]^. It has also been exhibited that GNT hinders the growth of LAPC-4 and PC3 cells in a dose-dependent manner^[^^14^^]^. GNT is an inhibitor of protein kinases (except tyrosine-protein kinase, p40), histidine protein kinase, and 5α-reductase^[^^15^^-^^17^^]^. 

Cell-based experiments are used as a significant method in the process of discovering new drugs. Cell culture is also considered as an important laboratory process in drug discovery and development. Owing to the limitations of conventional 2D culture, the 3D cell culture methods are suggested as a novel technique to produce more accurate findings^[^^18^^,^^19^^]^. Moreover, the 3D cell culture offers more in cell-cell and cell-extracellular matrix interactions compared to 2D cultures^[^^20^^]^. Therefore, in this study, we assessed the anticancer effects of GNT on PC3 cell lines under a 3D culture medium. 

## MATERIALS AND METHODS

**Materials**

DMEM/F12 culture medium and FBS was provided by Gibco (USA), Trypsin, EDTA, penicillin-streptomycin, Triton X-100, red phenol, and trypan blue were purchased from Sigma (USA). DMSO solution was obtained from Scharlau (Spain), MTT powder from Sisco Research Laboratories (India), neutral red powder, sodium hydroxide, hydrochloric acid, ethidium bromide, acetic acid, agarose, and phosphoric acid were procured from Merck (Germany). Both 24- and 96- well plates were acquired from Orange Scientific (Belgium). Other materials were sourced from local *companies.*

**Cytotoxic evaluation of GNT**

The Xi'an Saiyang Biotechnology Company in China provided GNT with 98% purity. To remove biological contaminants from GNT, we incubated 0.02 mg of GNT with sterile DMEM containing 1% antibiotic-antimycotic (1 ml) in a CO_2_ incubator (37 °C and 5% CO_2_). After overnight incubation, media were discarded, and fresh media containing different concentrations of GNT (120, 240, and 480 µM) were prepared. In the end, the cytotoxicity of GNT on PCa cells (PC3), supplied from the Pasteur Institute of Iran (Tehran), was evaluated.

**Three-dimensional culture of PC3 cells**

**Fig. 1:**
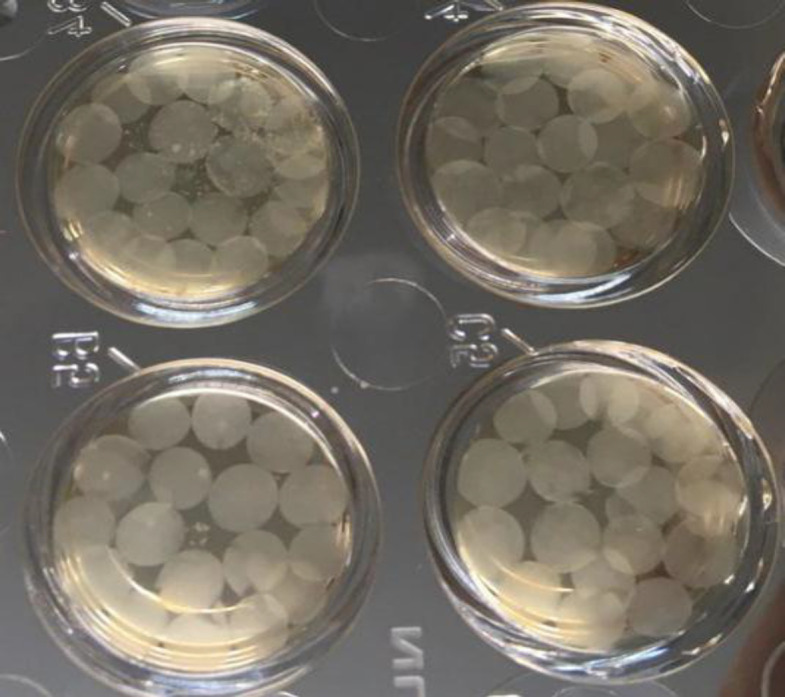
Polymerized alginate capsules containing PC3 cells in plate wells containing culture medium

Cell encapsulation technique in alginate hydrogel was used to create a 3D culture medium. To this end, 0.11 g of sodium alginate powder was dissolved in 10 ml of 0.9% sodium chloride solution to prepare an alginate solution. The solution was filtered by a 0.22 μm plastic syringe and added to the cell plate containing 2 × 10^6^ of PC3 cells and re-suspended. Cell individualization and suspension in alginate solution were performed using a 22G plastic syringe. Alginate capsules were produced by injecting the cell-alginate mixture into a bath of 100 mM of calcium chloride; drops were released into the bath from a distance of 5 cm above the bath's surface. All the capsules were allowed to be polymerized for 10 minutes (Fig. 1). In the next step, after removal of calcium chloride, the capsules were washed three times with PBS (pH 7.4), and the cells were rinsed once again with 1 ml of DMEM solution containing 10% FBS. Finally, the capsules comprising PC3 cells were incubated in 80% humidity and 5% CO_2_ at 37 °C for 24 hours. For depolymerization of capsules, after removing the media, the capsules were transferred to sterile tubes and washed three times with PBS. Then 1 ml of depolymerization solution (50 mM of sodium citrate) was added to the cells^[^^21^^]^. 

**Treatment of cells with GNT**

A 96-well plate was seeded with a certain number of encapsulated PC3 cells in each assay. These cells were then incubated in 80% humidity and 5% CO_2 _at 37 °C overnight. Then culture medium in each well was replaced with a serum-free culture medium containing various concentrations of GNT (120, 240, and 480 µM) and further kept in an incubator for 24 hours under the same incubation conditions stated above. 

**MTT assay**

Encapsulated PC3 cells (4 × 10^4^) were seeded into a 96-well plate and incubated in 80% humidity and 5% CO_2_ at 37 °C overnight. Subsequently, the old culture medium was replaced with a free-serum culture medium containing various concentrations of GNT (120, 240, and 480 µM) and then incubated for further 24 hours under the same above-mentioned conditions. Thereafter, 5 mg/ml of MTT was added to each well and incubated in the dark at 37 °C for 3 h. Finally, 200 μl of DMSO was added to each well and placed in a shaker incubator under dark conditions for 3 h. Following the complete dilution of MTT solution and release of the solution into the capsules, 100 μl of the solution was removed, and light absorption of each well was measured at 570 nm using a plate reader (Biotek, USA). In this assay, the culture medium was used as the negative control, and the cells containing different concentrations of GNT (120, 240, and 480 µM) was used as the treated samples. MTT assay was repeated three times for each concentration of GNT.

**Neutral red uptake assay**

To further confirm the findings of the MTT assay, we performed neutral red absorption technique^[^^22^^]^. For this purpose, 4 × 10^4^ of encapsulated PC3 cells were incubated in 80% humidity and 5% CO_2_ at 37 °C overnight. The old cell culture medium was removed from each well, and a new free-serum culture medium containing varying concentrations of GNT (120, 240, and 480 µM) was added and incubated for further 24 hours under the same incubation conditions mentioned above. Each well received 4 µl of neutral red solution at a concentration of 5 mg/ml. The plate was then incubated in the dark in 80% humidity and 5% CO_2_ at 37 °C until the formation of red crystals. Afterwards, the supernatant was discarded and cells were washed twice with PBS. Then 200 μl of fixation buffer (formaldehyde 37% (v/v), CaCl2 (10% (w/v) in water) was added to each well and incubated for 1 min. Subsequently, 100 μl of the solubilizing buffer (acetic acid 5%) was added and incubated in a shaker incubator in darkness for 20 min. Finally, a plate reader (Biotech, USA) was used to determine the light absorption of each well at 540 nm. This experiment was carried out three times for each concentration of GNT. 

**Comet assay**

A useful method for determination of DNA strand breaking is the comet assay or single cell gel electrophoresis assay^[^^23^^]^. In a 3D culture medium, the effect of GNT concentrations (120, 240, and 480 µM) on the PC3 cell viability was evaluated. After encapsulating 12 × 10^4 ^of PC3 cells and incubating in 80% humidity and 5% CO_2_ at 37 °C overnight, the initial culture medium was discarded and 300 µl of the fresh culture media (free-serum) containing various amounts of GNT was added. The cell-containing plate was then incubated in an incubator for 24 hours. Thereafter, the capsules containing the cells were collected separately from each well and transferred to 1.5-ml tubes. Dissolution of alginate capsules was based on a previously described method^[^^21^^]^. Finally, 200 µl of PBS was added to each test tube, and the cells were separated and isolated using a needle. For the alkaline comet test, the slides were covered with agarose with a normal melting point and refrigerated at 4 °C for 10 minutes. Next, the cell suspension was mixed in a 1:2 ratio with low melting point agarose and then poured onto slides. A coverslip was placed on each slide and stored in the refrigerator for 10 minutes to create a layer of cells. To perform cell lubrication and nucleus distraction, all slides were incubated in a cold lubricating buffer (NaCl, 2.5M; EDTA, 100 mM; Tris, 10 mM; NaOH, 0.2 M; and Triton X-100, %1; pH 10) at 4 °C for 16-18 h. To unzip the DNA, slides were washed twice with electrophoresis buffer at 4 °C for 40 min and electrophoresed at 25 V and 300 mA at 4°C for 45 min. The slides were put in a neutralization buffer (0.04 M of Tris, pH 7.5) for 10 minutes to neutralize the basic environment. They were then treated with 100 µl of ethidium bromide solution at a concentration of 20 µg/ml at room temperature for 10 minutes. After staining and washing twice with distilled water for 10 minutes, the slides were evaluated under a fluorescence microscope (Nikon, Japan), and results were statistically analyzed.

**Cytochrome C assay**

In each well of six-well plate, 1 × 10^6 ^of encapsulated PC3 cells were cultured and incubated in 80% humidity and 5% CO_2_ at 37 °C overnight. Afterwards, the old culture medium was removed from each well and replaced with a free-serum culture medium containing varied concentrations of GNT (120, 240, and 480 µM), which was then placed back in the incubator for 24 hours under the same incubation conditions mentioned above. After transferring to the 1.5-mL tubes, the cells were dissolved in 1 mL of cytosolic extraction buffer and incubated on ice for 10 minutes. The cells were then homogenized in a Dounce tissue grinder and transferred to new 1.5-ml tubes, where they were centrifuged at 700 ×g at 4 °C for 10 minutes. The supernatants of the samples were then transferred to the fresh tubes and further centrifuged at 10,000 ×g at 4 °C for 30 minutes. The soluble samples, which was the cytosolic component, was collected. Protein concentration of the treated PC3 cells was measured using the Bradford protein assay. 

**NO** **determination**

Oxidative stress has been shown to trigger apoptosis in cancer cells^[^^24]^. Similar to MTT assay, cells were first counted (5 × 10^5^ per 96-well plate) and various concentrations of GNT (120, 240, and 480 µM) were added to the wells and incubated. Encapsulated PC3 cells were exposed to 120, 240, and 480 µM concentrations of GNT for 24 h. Thereafter, the media from each well were transferred to sterile tubes and centrifuged at 500 ×g at 4 °C for 5 minutes. A total volume of the media was transferred to a 96-wells plate and mixed with 100 µl of Griess reagent (Sigma; 0.04 g/ml in PBS, pH 7.4) and incubated at room temperature for 10 minutes. Absorbance was measured by a Biotech Eliza reader at 520-550 nm for 30 minutes. The NO concentration in the treated PC3 cells was calculated using the sodium nitrite standard curve. 

**GSH determination assay**

The process of counting cells in 24-well plates was similar to the NO assay (5 × 10^5^/well). Encapsulated PC3 cells were transferred to a 24-well plate and incubated in 5% CO_2_ at 37 °C overnight. Then the old media were discarded, and fresh media containing 120, 240, and 480 μM of GNT were added to each well and incubated at 37 °C for 24 h. Next, 200 µl of the lubricating buffer was added to each well, and protein concentration was assessed by the Bradford assay. About 40 μl of the obtained solution was removed and transferred to new tubes, to which 40 µl of 10% trichloroacetic acid solution was added and then stored at 4 °C for 2 hours. Centrifugation was performed at 1500 ×g for 15 minutes. The solution was then removed from the top of the tubes, and 75 µl of the lubricating buffer, 55 µl of Tris HCl buffer (pH 8.5), and 25 µl of dinitrothiocyanobenzene were added to each tube. Finally, the absorbance of samples was measured at 412 nm.

**Catalase activity assay**

The steps of the catalase test were similar to the GSH test. Briefly, 5 µl of samples were mixed with 50 µl of lubricating buffer, 20 µl of distilled water, and 25 µl of 15% hydrogen peroxide were added to each tube, and the tubes (capsules) were placed on a shaker. After a 2-min incubation period at 37 °C, the tubes were mixed with 100 µl of potassium dichromate solution. At this stage, the pink color of the solution, and the blue color of the upper part of solution were visible. Finally, the tubes were incubated at 100 °C for 10-15 minutes until the color changed to green. The absorbance was measured at 570 nm using a plate reader (Biotech). 

**Statistical analysis**

Each test was repeated three times, and the obtained results were reported as mean ± SD. Statistical analysis of the obtained data was performed using GraphPad InStat software (version 6). Comparison of the results was analyzed by a two-way analysis of variance (ANOVA). *p *< 0.05 was considered statistically significant.

## RESULTS

**Viability of PC3 cell in the presence of GNT**

**Fig. 2:**
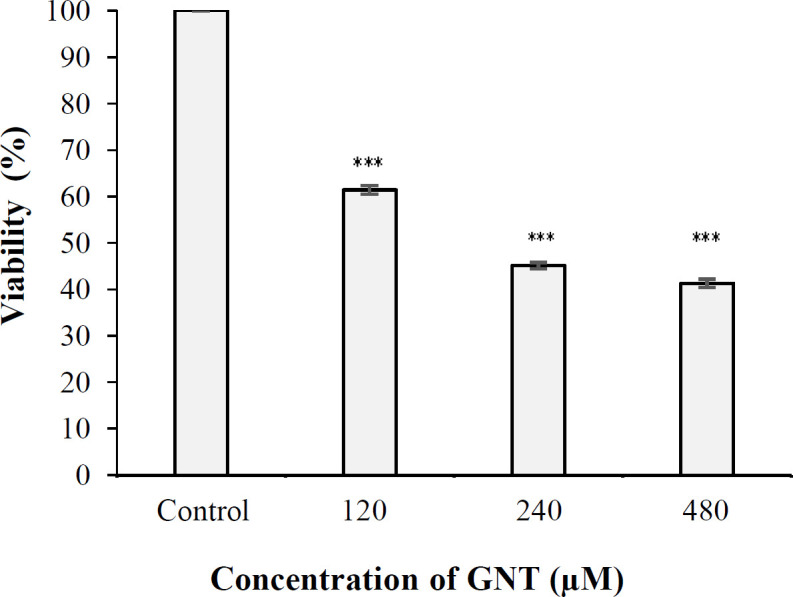
Viability of PC3 cells in 3D culture after 24 hours of exposure to 120, 240, and 480 μM of GNT (^***^p <0.001 when compared to the control)

In the present study, the impact of GNT at the concentrations of 120, 240, and 480 µM on the growth rate of PC3 cells was assessed *in vitro*. Culture medium containing PC3 cells was used as the negative control. The percentage of cell viability in the presence of GNT was determined using the MTT assay. The results demonstrated that viability of PC3 cell in 3D culture was 61.4%, 45.1%, and 41.3% in the presence of 120, 240, and 480 µM of GNT, respectively. The results of the MTT reduction assay showed that IC_50_ of GNT was 480 μM after 24 hours of incubation. The results also showed that the inhibitory impact of GNT on PCa cells was significant in all three concentrations compared to the control (*p* < 0.001), and that increasing concentration of GNT decreased the growth of PCa cells (Fig. 2). Also, cell viability was much higher at 120 μM than 240 and 480 μM, and the difference in the percentage of cell viability between 240 and 480 µM concentrations of GNT was statistically significant (*p* < 0.001). 

**Results of** **neutral** **red uptake assay**

**Fig. 3:**
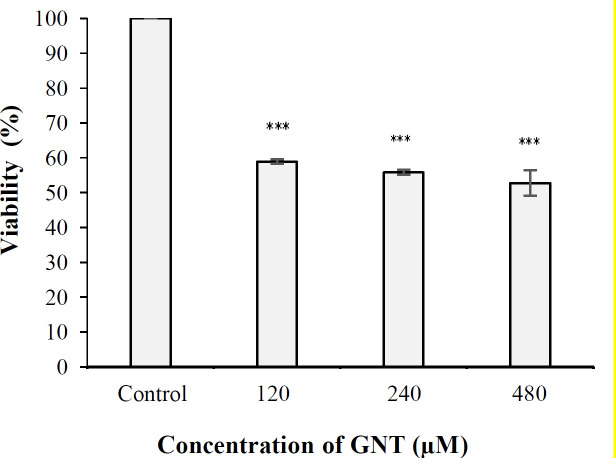
Viability percentage of PC3 cells in 3D culture after 24 hours of exposure to 120, 240, and 480 μM of GNT (^***^p < 0.001 when compared to the control)

Findings of the neutral red assay verified the results of the MTT assay, indicating that the percentage of PC3 cell viability was 58%, 55%, and 52% at concentrations of 120, 240, and 480 µM of GNT, respectively. The results also indicated that the inhibitory effect of GNT on PCa cells growth was significant at all three concentrations compared to the control sample (cells cultured in alginate capsules without GNT) (Fig. 3). Statistical analysis also revealed that the cell viability was insignificant at 120 µM compared to 240 and 480 µM and also 240 µM compared to 480 µM of GNT (*p* > 0.05).

**Comet assay results**

**Fig. 4:**
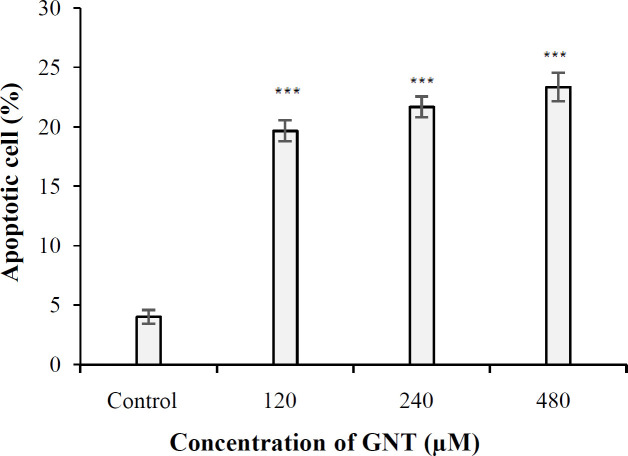
The rate of apoptosis in PC3 cells at different concentrations of GNT in 3D culture, based on comet assay (^***^p < 0.001)

The aim of the alkaline comet assay is to investigate the role of GNT in inducing cell cytotoxicity via apoptosis induction in PC3 cells. The results showed that the amount of apoptosis induction in the control sample and the samples treated with 120, 240, and 480 μM of GNT was 4%, 19.66%, 21.66%, and 23.33%, respectively. The proportion of apoptosis was significant for all concentrations of GNT compared to the control (Fig. 4). Furthermore, there was a significant difference between the percentage of apoptosis in the cell treated with three concentrations of 120, 240, and 480 μM of GNT (*p* ≤ 0.001). 

**Cytochrome C assay results**

**Fig. 5:**
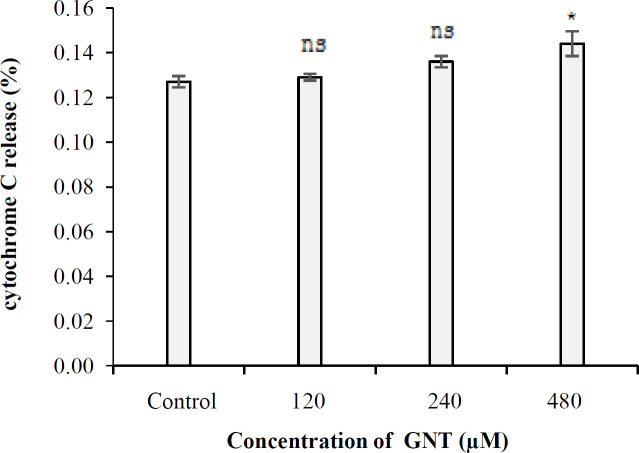
The cytochrome C released into the cytosol of PC3 cells at different concentrations of GNT in 3D culture. ^*^p < 0.05, compared to the control. ns, not significant

In 3D culture, the amount of cytochrome c inside the PC3 cells was 0.127%, 0.129%, 0.136%, and 0.149%, in the control samples and the samples treated with 120, 240, and 480 µM concentrations of GNT, respectively. As shown in Figure 5, the level of cytochrome C in the samples treated with 120 and 240 µM of GNT was not significantly different from that of the control (*p *> 0.05), but for 480 µM, a significant difference was observed (*p* < 0.05). Furthermore, no significant difference was found between the amount of cytochrome C in GNT-treated samples (120 µM vs. 240 µM and 240 µM vs. 480 μM; *p* > 0.05). 

**Results of** **NO assay**

**Fig. 6:**
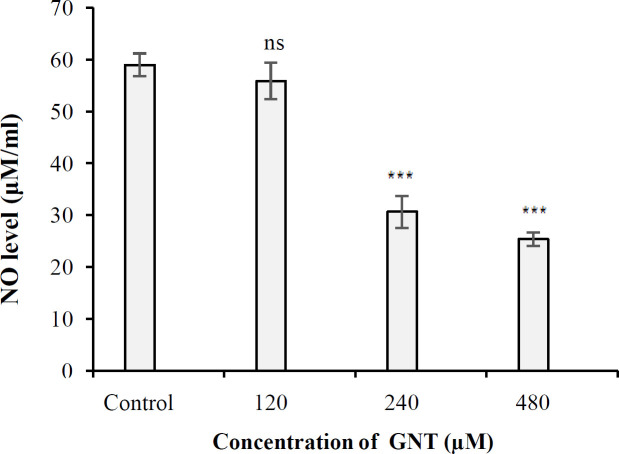
The amount of NO released from PC3 cells in 3D culture in the presence of 120, 240, and 480 μM concentrations of GNT. ^***^p < 0.001 compared to the control. ns, not significant

The NO released from PC3 PCa cells under 3D culture conditions in the control and also 120, 240, and 480 μM treated-samples were 58.98, 55.88, 30.62 and 25.37 μM/ml, respectively. The amount of NO released from the cells due to the effect of GNT in all concentrations except 120 μM was significant compared to the control (Fig. 6). As a result, GNT significantly decreased the amount of NO in PC3 cells in 3D culture compared to the control. 

**GSH assay results**

**Fig.7:**
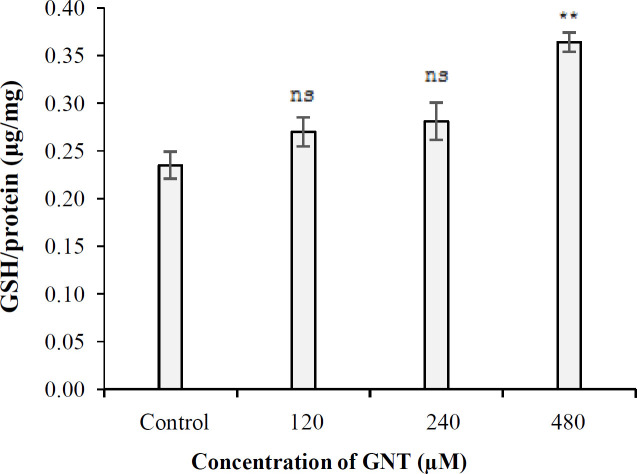
The level of GSH in PC3 cells in the presence of different concentrations of GNT in 3D culture. ^**^p < 0.01, compared to the control. ns, not significant

Under 3D culture conditions, the level of cellular GSH generated in the control samples and the samples treated with 120, 240, and 480 µM of GNT were 0.235, 0.270, 0.282, and 0.364 GSH (μg)/protein (mg) respectively. The amount of GSH in the samples treated with 120 and 240 µM of GNT was not significantly different (*p* > 0.05) compared to the control (Fig. 7); however, at 480 µM concentration, a significant difference was observed in the level of GSH compared to control (*p* < 0.01).

**Results of** **catalase activity assay**

**Fig. 8:**
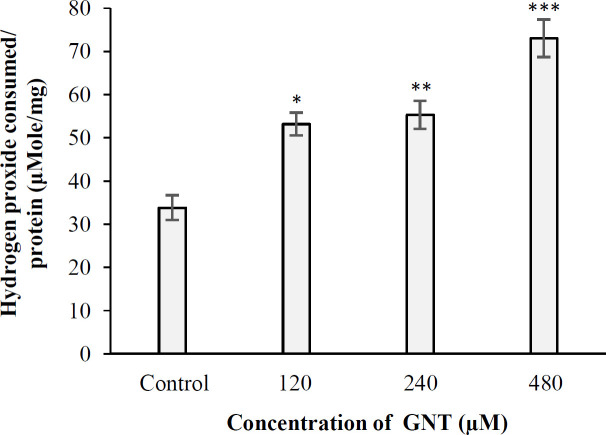
The amount of catalase produced in PC3 cells following treating with different concentrations of GNT in 3D culture (^***^p < 0.001, ^**^p < 0.01, ^*^P < 0.05 compared to the control)

In this experiment, the level of cellular catalase under 3D culture conditions in the control samples and the samples treated with 120, 240, and 480 µM of GNT were found to be 33.81, 53.15, 55.28, and 72.99 μmole of hydrogen peroxide consumed/min/mg protein, respectively. In all concentrations of GNT, the amount of catalase produced was significant compared to the control (Fig. 8). Furthermore, there was a significant difference in terms of catalase level between the samples treated with 120 and 480 µM and also 240 and 480 μM (*p* < 0.05).

## DISCUSSION

Cancer is one of the major causes of mortality across the world, and the number of cancer cases is on the rise^[^^4^^]^. The main purpose of cancer therapy is to remove tumor, prevent metastasis, improve patients' survival and promote quality of life. Given the toxicity of conventional therapies, researchers have sought for a secondary compound that could enhance the potential of cancer therapy, while lowering its toxicity^[^^25^^]^. PCa is one of the most frequent cancers and the second major cause of cancer mortality in men^[^^26^^]^. Herbs and active compounds derived from medicinal plants are now being investigated as a supplement for the treatment of several forms of cancer. Recent scientific reports have shown that GNT has anticancer effects on breast^[^^27^^]^, colon^[^^28^^]^, lung^[^^29^^]^, stomatch^[^^30^^]^, and prostate^[^^31^^]^ tissues in a 2D culture medium. However, in many cases, the toxicological results of 2D cell culture are not very reliable for use in human experiments; therefore, 3D cell culture studies have been suggested to produce more accurate results and also to reduce the cost of research^[^^32^^]^.

Based on the literature survey, the addition of GNT to LNCaP PCa cells at concentrations more than 10 µM suppressed PCa cell growth for three days^[^^33^^]^. The flow cytometric evaluation of PCa cells treated with 50 µM of GNT also showed that the number of cells in the G2/M phase increased from 6.6% to 27.1% after 24 hours and reached 43.5% after 96 hours. The TUNEL test was used to assess the percentage of cells undergone apoptosis. The number of cells with apoptosis raised from 6.65% to 16.42% after 72 hours of administration of 50 µM of GNT^[^^33^^]^.

In 2010, Hsu *et al.*^[^^31^^]^ studied the anticancer effects of GNT on PC3 PCa cells and demonstrated that GNT inhibits cancer cell growth by stopping the cell cycle and inducing apoptosis. Sanaei *et al.*^[^^34^^]^ used flow cytometry to assess the influence of GNT on the rate of induction of apoptosis in hepatocellular carcinoma Hepa1-6 cells. Their results demonstrated that GNT prevented the growth of cancer cells via apoptosis. In addition, after 24, 48, and 72 hours of treatment with 20 µM of GNT, the percentage of apoptotic cells was 35%, 42%, and 65%, respectively. They also concluded that the rate of apoptosis induction is dependent on the quantity of GNT and the length of exposure time of the cells to this compound. Shafiee and colleagues^[^^25^^]^ studied the effects of GNT in the suppression of proliferation, inhibition of metastasis, and induction of apoptosis in PC3 PCa cells using 30, 50, and 70 mM concentrations of GNT. The clonogenic assay revealed that increasing the concentration of GNT could reduce the cancer cell proliferation. Furthermore, GNT could strongly suppressed metastatic potency of PC3 PCa cells and enhanced apoptosis induction via activating caspase-3 pathways. They also observed the maximum cytotoxicity at 50 mM of GNT^[^^25^^]^.

In the present study, after preparing GNT with 98% purity, the method of encapsulating cells in alginate hydrogel was used to achieve a 3D environment. To evaluate the cytotoxic and anticancer effects of GNT at concentrations of 120, 240, and 480 µM in the 3D culture, MTT, neutral red, cytochrome C, and comet assays were used. In addition, determination of NO, catalase, and GSH levels were performed to evaluate the effect of GNT on the cellular stress. The results of the MTT assay showed that IC_50_ of GNT was 480 μM after 24 hours of incubation with PCa cells. The results of cytotoxic effect of GNT on PC3 cells under 3D culture conditions demonstrated that the cytotoxicity rate of cells treated with the above concentrations was ascending (42, 45, and 48, respectively). The anticancer effects of GNT on PCa cells in the 3D culture are consistent with the findings of the studies conducted in 2D cell culture conditions, and there is only a variation in the amounts of GNT used. An important reason for using a higher concentration of GNT in this study is related to the nature of the cell culture in 3D conditions. The concentration of materials in 3D culture may decrease by increasing the depth of cell placement in the scaffold^[^^35^^,^^36^^]^. One of the benefits of 3D culture is that the settings are remarkably similar to those in which cancer cell develop in the living body^[^^37^^]^. Based on the comet assay results, the rate of apoptosis induction in the control sample and the cells treated with 120, 240, and 480 µM of GNT exhibited a dose-dependent rising trend and was 4%, 19.66%, 21.66%, and 23.33%, respectively. This finding is consistent with those of Hsu *et al.*^[^^31^^]^, Sanaei *et al.*^[^^34^^]^, and Shafiee *et al.*^[^^25^^]^. 

The mitochondrial intermembrane space contains a heterogeneous class of proteins whose release causes cell death. The first molecule belonging to this class of proapoptotic proteins that has been identified at the molecular level is Cytochrome c^[^^38^^]^. The amount of cytochrome C released into the cytosol of PC3 cells under 3D growth conditions was 0.127%, 0.129%, 0.136%, and 0.149% in the control cells and the cells treated with 120, 240, and 480 µM of GNT, respectively. As a result, the level of cytochrome C in PC3 cells treated with different concentrations of GNT was not significantly different from that of the control (*p* > 0.05). Based on these findings, it could be deduced that apoptosis occurs through a nonmitochondrial pathway. Effect of GNT on the NO generation in 3D culture conditions was investigated for the first time in this study, and catalase and GSH assays were used to corroborate the results of the NO test. These experiments found that GNT did not trigger the synthesis of NO at the concentrations tested, but increasing the concentration of GNT led to the enhanced production of catalase when compared to the control. However, the quantity of cellular GSH was not significantly different from the control at all concentrations used, except for 480 µM. Based on the above-mentioned observations, GNT appears to have limited antioxidant effect on PCa cells. 

Collectively, our results demonstrated that GNT in 3D culture conditions causes mortality in PC3 cells. Comparing the MTT with alkaline comet results suggested that GNT reduces cancer cell viability by inducing apoptosis. In addition, due to the nonsignificant amount of cytochrome c released from mitochondria in cells treated with different concentrations of GNT, this molecule induced apoptosis in cancer cells through the nonmitochondrial pathway. The findings of this study disclose for the first time that GNT has ability to reduce the production of cellular NO and increase catalase and glutathione production. GNT seems to have anticancer properties and few antioxidant capabilities and could cause the death of PCa cells, mostly through triggering apoptosis.

## DECLARATIONS

### Acknowledgments

This article has been derived from a Ph.D. thesis (number: 162519957) at Department of Medical Radiation Engineering, Central Tehran Branch, Islamic Azad University (Tehran, Iran). We would like to thank the cooperation of all members of the Imam Hossain University for their assistance. 

### Ethical statement

Not applicable.

### Data availability

The analyzed data sets generated during the study are available from the corresponding author on reasonable request.

### Author contributions

SMK: study concept and design, acquisition of data, drafting of the manuscript, critical revision of the manuscript for important intellectual content and statistical analysis; MSB: study concept and design, acquisition of data, and statistical analysis; JZ: study concept and design, analysis and interpretation of data, statistical analysis, and administrative, technical, and material support; MS: analysis and interpretation of data; NB: study concept and design and statistical analysis; AHN: critical revision of the manuscript for important intellectual content and administrative, technical, and material support; HKA: critical revision of the manuscript for important intellectual content and administrative, technical, and material support; MM: critical revision of the manuscript for important intellectual content and administrative, technical, and material support.

### Conflict of interest

The authors declare that they have no conflicts interest.

### Funding/support

This research did not receive any specific grant from funding agencies in the public, commercial, or not for profit sectors.
